# Investigation of Lipid Profile and Clinical Manifestations in SCA Children

**DOI:** 10.1155/2020/8842362

**Published:** 2020-08-18

**Authors:** Caroline Conceição da Guarda, Sètondji Cocou Modeste Alexandre Yahouédéhou, Rayra Pereira Santiago, Camila Felix de Lima Fernandes, Joelma Santana dos Santos Neres, Antonio Mateus de Jesus Oliveira, Milena Magalhães Aleluia, Camylla Vilas Boas Figueiredo, Cleverson Alves Fonseca, Luciana Magalhães Fiuza, Suellen Pinheiro Carvalho, Rodrigo Mota de Oliveira, Valma Maria Lopes Nascimento, Larissa Carneiro Rocha, Marilda Souza Gonçalves

**Affiliations:** ^1^Laboratório de Investigação em Genética e Hematologia Translacional, Instituto Gonçalo Moniz, FIOCRUZ-BA, Salvador, Bahia, Brazil; ^2^Departamento de Ciências Biológicas, Universidade Estadual de Santa Cruz (UESC), Bahia, Brazil; ^3^Laboratório de Pesquisa em Anemias, Departamento de Análises Clínicas e Toxicológicas, Faculdade de Farmácia, Universidade Federal da Bahia, Salvador, Bahia, Brazil; ^4^Fundação de Hematologia e Hemoterapia do Estado da Bahia (HEMOBA), Salvador, Bahia, Brazil

## Abstract

**Introduction:**

Clinical complications in sickle cell anemia (SCA) are heterogeneous and involve several molecules. It has been suggested that SCA individuals present a dyslipidemic phenotype and that lipid parameters are associated with severe clinical complications, such as pulmonary hypertension. We sought to investigate associations between lipid parameters and clinical manifestations, as well as other laboratory parameters in a population of pediatric SCA patients.

**Methods:**

Our cross-sectional evaluation included 126 SCA patients in steady state and who were not undergoing lipid-lowering therapy. Hematological and biochemical parameters were characterized, and previous clinical manifestations were investigated.

**Results:**

Total cholesterol and low-density lipoprotein cholesterol (LDL-C) levels were increased in patients with a previous history of pneumonia, which also positively correlated with HbS levels. Decreased LDL-C levels were also associated with leg ulcers and anemia. Elevated high-density lipoprotein cholesterol (HDL-C) levels were associated with pain crises, increased viscosity, and decreased hemolysis. Several studies have determined that lipids play a role in the vascular impairment seen in SCA, which was corroborated by our findings.

**Conclusions:**

In sum, our results suggest that total cholesterol, HDL-C, and LDL-C levels are associated with hemolysis and anemia markers and, most importantly, with clinical complications related to vasculopathy in SCA.

## 1. Introduction

The single point mutation in sickle cell anemia (SCA) is responsible for the production of the variant hemoglobin S (HbS) which under low oxygen tension forms long polymers affecting the red cell morphology [1]. HbS polymerization is the first pathophysiological step leading to clinical manifestations in SCA; moreover, several different mechanisms are involved in the pathogenesis of the disease including ischemia reperfusion injury [2]; increased adhesiveness of leukocytes, reticulocytes, and endothelial cells culminating in vasoocclusion (VO) [3]; and the innate immune system activation with hemolysis products, known as erythrocyte damage-associated molecular pattern molecules (eDAMPs) [4]. Intravascular hemolysis and VO are hallmarks of SCA. Red blood cell lysis releases arginase, free heme, and hemoglobin which decreases the L-arginine pool, the main source for endothelial cells to produce nitric oxide (NO), and leads to endothelial dysfunction and VO [1]. In addition, many inflammatory molecules have been described in SCA such as cytokines, chemokines, adhesion molecules, NO, heme, reactive oxygen species, adenosine triphosphate (ATP), and lipid mediators [5–7].

Cholesterol is obtained from the diet or may be produced endogenously and is the precursor of steroid hormones, bile acids, and vitamin D and contributes to cell membrane fluidity [8]. It is transported to the tissues packaged with apolipoproteins, therefore generating the blood lipoproteins chylomicrons (CM), very low-density lipoprotein cholesterol (VLDL-C), low-density lipoprotein cholesterol (LDL-C), and high-density lipoprotein cholesterol (HDL-C) [8, 9]. Abnormal lipid homeostasis is related to different inflammatory diseases, including Alzheimer, where alterations in sphingolipid and cholesterol metabolism result in accumulation of long-chain ceramides and cholesterol [10], and psoriasis, since patients present significantly higher cholesterol levels in the VLDL-C and HDL-C fractions [11]. It is thought that LDL-C plays an important proinflammatory role in vascular diseases, while HDL-C is thought to be anti-inflammatory depending on the context [12].

Moreover, altered lipid parameters are directly and mostly associated to the development of cardiovascular diseases [13], due to the relevance of several cohort studies which have shown that elevations of plasma LDL-C levels in association with decreased HDL-C levels consist in an important risk factor for atherosclerosis and other vascular complications [14]. In atherosclerosis, several pathophysiological mechanisms are similar to SCA vasculopathy such as decreased NO bioavailability, oxidative stress, and endothelial dysfunction [15], although the formation of atheroma plaques is not observed in SCA. Vasculopathy in SCA is closely related to complications such as pulmonary hypertension, leg ulceration, priapism, and stroke [16].

It has been over 40 years since the first study suggesting a relationship of lipid determinations and laboratory parameters or clinical manifestations in SCA was published [17]. Since then, several studies attempted to investigate the role of lipids in the pathophysiological mechanism of SCA. It was shown that SCA individuals present decreased total cholesterol levels as well as HDL-C and LDL-C in addition to increased triglycerides and VLDL-C levels [18–20].

Clinical complications in SCA are heterogeneous and often associated to hemolysis such as pulmonary hypertension, leg ulcers, and priapism and to VO such as vasoocclusive pain crises, acute chest syndrome, and osteonecrosis, suggesting two subphenotypes [16]. However, there is evidence that pulmonary hypertension, for instance, seems to be associated to hemolysis, VO, and elevated triglyceride levels in a large cohort of individuals with sickle cell disease (SCD) with increased tricuspid regurgitant velocity [20]. In addition, during vasoocclusive crises, patients with SCA presented total cholesterol, triglyceride, and LDL-C levels significantly decreased whereas HDL-C levels were increased when compared to steady state [21]. Also, during steady state, HDL-C levels were found to be associated to nitric oxide metabolites (NOm) and fetal hemoglobin (HbF) levels [18], two of the most important prognostic biomarkers in SCA.

Considering the vascular involvement of both pulmonary hypertension and vasoocclusive crises, the combined data indicate that lipid parameters may be associated to vasculopathy in SCA. Therefore, we attempted to investigate the association of lipid profile (total cholesterol, LDL-C, and HDL-C) with clinical complications and laboratory measurement of hemolysis, anemia, hemoglobin S (HbS), and systemic NO.

## 2. Methods

### 2.1. Study Design and Patients

A cross-sectional study was performed including 126 SCA individuals (homozygous HbSS genotype), all seen at the Bahia Hemotherapy and Hematology Foundation (HEMOBA), located in Salvador, Bahia, Brazil. The mean patient age was 14.5 ± 3.5 years; in addition, median patient age was 15 years (IQR 12-17 years) and 60 (47.6%) were female. Patients with SCA in steady state, defined as the absence of acute episodes in the past three months, were recruited to participate during routine clinical visits. All patients were taking folic acid supplementation, 60 were taking hydroxyurea, and none were undergoing therapy with lipid-lowering agents, such as statins. Data regarding the occurrence of previous clinical manifestations was collected using a standardized and confidential questionnaire (self-reported or reported by a legal guardian of the patient) at the time of enrollment and subsequently confirmed by medical records. The present study was approved by the Institutional Research Board of the São Rafael Hospital (protocol number 1400535) and was conducted in compliance with the ethical principles established by the Declaration of Helsinki and its later revisions. All patients were informed regarding the purpose and procedures of this study, and informed written consent was obtained from each SCA patient's legal guardian.

### 2.2. Hematological Parameters

Blood samples were collected at the time of enrollment after a 12-hour fast and analyzed immediately. Hematological parameters, including complete blood counts, were examined using a Beckman Coulter LH 780 Hematology Analyzer (Beckman Coulter, Brea, California, USA), and blood smears were stained with Wright's stain and examined by optical light microscopy. Reticulocytes were counted after staining supravitally with brilliant cresyl blue dye. Hemoglobin genotyping was performed by high-performance liquid chromatography on an HPLC/Variant II hemoglobin testing system (Bio-Rad, Hercules, California, USA) to confirm the presence of HbSS.

### 2.3. Biochemical Determinations

LDL-C and VLDL-C levels were determined by the Friedewald equation [22], while total cholesterol, HDL-C, and triglycerides as well as biochemical parameters, including total bilirubin and fractions, lactate dehydrogenase, iron, and hepatic (aspartate aminotransferase, alanine aminotransferase, and gamma-glutamyl transferase) and renal (urea, creatinine and uric acid) markers, were measured in serum samples using an automated A25 chemistry analyzer (Biosystems S.A., Barcelona, Catalunya, Spain).

Ferritin levels were determined using an Access 2 Immunochemistry System (Beckman Coulter Inc., Pasadena, California, USA). NO metabolites (NOm) were quantified in serum samples with Griess reagent employing SoftMax Pro software, as previously described [23]. Laboratory analyses were performed at the Clinical and Toxicological Analysis Laboratory of the College of Pharmaceutical Sciences, Federal University of Bahia (LACTFAR-UFBA).

### 2.4. Statistical Analyses

Statistical analyses were performed using the Statistical Package for the Social Sciences (SPSS) version 20.0 software (IBM, Armonk, New York, USA) and GraphPad Prism version 6.0 (GraphPad Software, San Diego, California, USA), which was also used to assemble graphs. The clinical characteristics of the study participants are expressed as means and respective standard variations. The distribution of each variable was tested by employing the Shapiro-Wilk test. The Mann-Whitney *U* test was used when the variables presented a nonparametric distribution, and independent *t*-test was used when the variables presented a parametric distribution. Fisher's exact test was used to compare categorical variables. Spearman correlation rank analysis was performed to test correlations between lipid parameters and hematological parameters. *p* values < 0.05 were considered statistically significant.

## 3. Results

### 3.1. Investigation of Clinical Manifestations and Lipid Parameters in SCA

**Figure 1:**
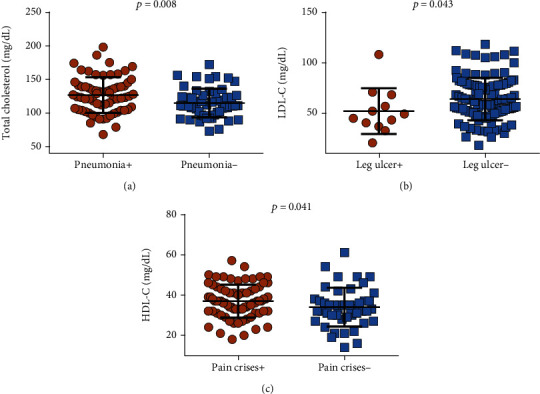
Associations between total cholesterol, LDL-C, and HDL-C levels and clinical manifestations in SCA. (a) Patients with a previous history of pneumonia (*N* = 69) exhibited increased total cholesterol levels. (b) Patients with a previous history of leg ulcers (*N* = 12) presented decreased LDL-C levels. (c) Patients with a previous history of pain crises (*N* = 78) had increased HDL-C levels. *p* values obtained using the Mann-Whitney *U* test.

We first decided to investigate associations between lipid parameters and previous clinical events using the Mann-Whitney *U* test. We found that patients with a previous history of pneumonia (postpneumonic) presented increased total cholesterol levels (Figure 1(a)), a previous history of leg ulcers was associated with decreased LDL-C levels (Figure 1(b)), and patients with a previous history of pain crises had increased HDL-C levels (Figure 1(c)).

### 3.2. Associations between Laboratory Parameters and Lipid Markers

In addition to clinical manifestations, we also investigated associations between laboratory and lipid parameters. The patients presented overall median total cholesterol levels of 118 mg/dL (IQR: 103.5–135.5 mg/dL) and median LDL-C levels of 58.8 mg/dL (IQR: 48.6–77.5 mg/dL). None of the patients presented total cholesterol levels higher than 200 mg/dL or LDL-C levels higher than 110 mg/dL, because of that we decided to use the median value.

The patients were then stratified considering each median lipid parameter value; moreover, regarding HDL-C, patients were stratified according to pathologically decreased (below 40 mg/dL) and normal HDL-C levels (over or equal to 40 mg/dL). Thus, association analyses were performed.

Patients with total cholesterol levels higher than the median value (≥118 mg/dL) were also found to present increased LDL-C, VLDL-C, triglyceride, and direct bilirubin levels, as well as decreased indirect bilirubin and HbF levels, in addition to basophil counts (Table 1).

Patients with higher HDL-C levels (≥40 mg/dL) also presented decreased red cell distribution width (RDW) and eosinophil counts as well as decreased VLDL-C, triglyceride, LDH, and NOm levels. In addition, they presented increased hemoglobin, hematocrit, total cholesterol, gamma-glutamyl transferase (GGT), ferritin, and HbF levels (Table 1).

Moreover, patients with higher LDL-C levels (≥58.8 mg/dL) also exhibited decreased mean corpuscular volume (MCV) and mean corpuscular hemoglobin (MCH) values (Table 1).

### 3.3. Associations between Clinical Manifestations and Lipid Parameters

As some clinical events were previously found to be associated with lipid parameters, we further investigated these associations in patients stratified according to each median lipid variable value. Patients with a previous history of pneumonia were found to exhibit total cholesterol (≥118 mg/dL) and LDL-C (≥58.8 mg/dL) levels higher than both median values (Table 2). No statistical significance was found regarding pain crises and leg ulcers.

### 3.4. Correlations between Lipid Markers and Laboratory Parameters

**Figure 2:**
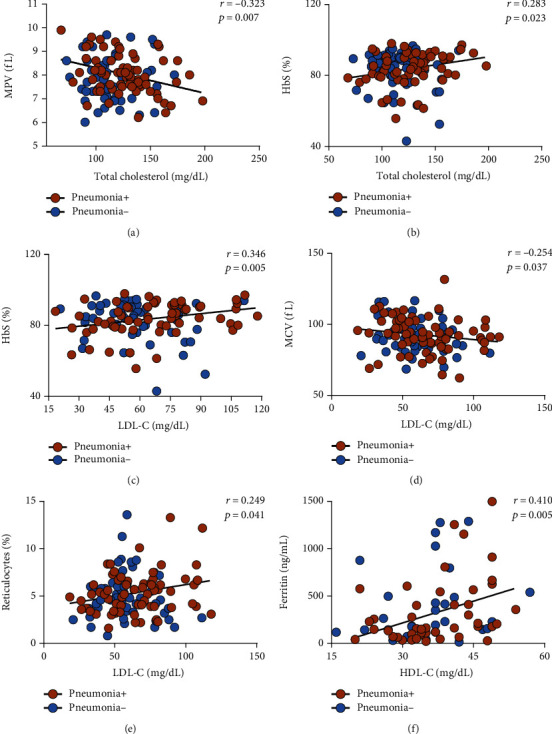
Correlations between lipid and hematological parameters in SCA patients with a previous history of pneumonia (*N* = 69) indicated by the brown circles. Patients without a previous history of pneumonia (*N* = 57) are indicated by the blue circles. (a) Total cholesterol levels were negatively correlated with mean platelet volume (MPV). (b) Total cholesterol levels were positively correlated with HbS levels. (c) LDL-C levels were positively correlated with HbS levels. (d) LDL-C levels were negatively correlated with MCV. (e) LDL-C levels were positively correlated with reticulocyte counts. (f) HDL-C levels were positively correlated with ferritin levels. Data comparisons made using Spearman's correlation rank test. None of the correlations described herein were statistically significant in patients without a previous history of pneumonia.

Correlation analysis was performed to investigate associations between laboratory and lipid parameters in patients with a previous history of pneumonia or pain crises. In patients with a previous history of pneumonia, total cholesterol levels were found to be negatively correlated with mean platelet volume (Figure 2(a)) and positively correlated with HbS (Figure 2(b)) levels. LDL-C levels were found to be positively correlated with HbS levels (Figure 2(c)), negatively correlated with mean corpuscular volume (Figure 2(d)), and positively correlated with reticulocyte counts (Figure 2(e)). HDL-C levels were positively correlated with ferritin levels (Figure 2(f)).

**Figure 3:**
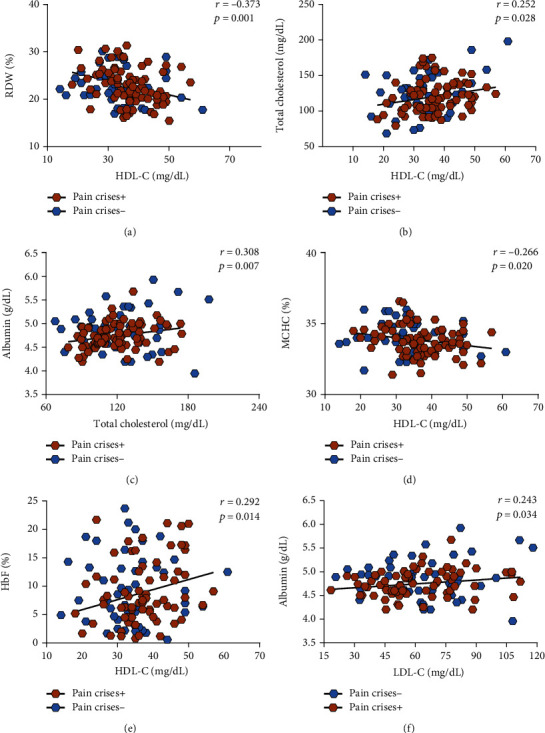
Correlations between HDL-C and triglyceride levels and hematological parameters in SCA patients with a previous history of pain crises (*N* = 78) indicated by the brown hexagons. Patients without a previous history of pain crises (*N* = 48) are indicated by the blue hexagons. (a) HDL-C levels were negatively correlated with RDW levels. (b) HDL-C levels were positively correlated with total cholesterol levels. (c) HDL-C levels were negatively correlated with HbF levels. (d) HDL-C levels were negatively correlated with MCHC. (e) Total cholesterol levels were positively correlated with albumin levels. (f) LDL-C levels were positively correlated with albumin levels. Data were compared using Spearman's correlation rank test. None of the correlations described herein were statistically significant in patients without a previous history of pain crises.

In patients with a previous history of pain crises, HDL-C levels were found to be negatively correlated with RDW (Figure 3(a)), positively correlated with total cholesterol levels (Figure 3(b)), and negatively correlated with HbF levels (Figure 3(c)) and MCHC (Figure 3(d)). Total cholesterol levels were found to be positively correlated with albumin levels (Figure 3(e)); in addition, LDL-C levels were also positively correlated with albumin levels (Figure 3(f)). It is relevant to highlight that these correlations were not statistically significant in patients without a previous history of pneumonia or pain crises.

## 4. Discussion

As has been previously demonstrated by several studies, the significant alterations in lipid parameters presented by SCA patients have been associated with hemolysis, anemia, vasoocclusive crises, activation of the TGF-*β* pathway, pulmonary hypertension, acute chest syndrome, and other complications [18–21, 24, 25]. Thus, we decided to investigate associations between the lipid profile in SCA individuals and previous history of clinical manifestations.

We found that SCA patients with increased total cholesterol levels (yet within physiological range) also had increased levels of LDL-C, VLDL-C, and triglycerides, as well as decreased levels of indirect bilirubin and HbF and a previous history of pneumonia. In postpneumonic patients, total cholesterol levels were also correlated with HbS and MPV, while LDL-C levels were correlated with HbS, MCV, and xferritin levels as well as reticulocyte counts. Pulmonary complications in SCA are mostly associated with vascular impairment and vasoconstriction, leading to VO. The occurrence of pneumonia in SCA patients has been closely linked to an increased frequency of acute chest syndrome, since the presentation of these conditions may overlap, and both are usually associated with pulmonary fat embolism and infectious pathogens [26]. In SCA patients, increasing levels of triglycerides were associated with more frequent episodes of acute chest syndrome [27]. Moreover, in a large cohort of adults, several associations were found between serum increased cholesterol levels and an increased risk of hospitalization leading to death due to respiratory disease [28]. The present study found a positive correlation between HbS levels and levels of total cholesterol and LDL-C in SCA patients with a previous history of pneumonia, which suggests that altered lipid parameters may indicate a more severe disease phenotype. Additionally, another study investigating lipoproteins in patients with SCD found a positive correlation between total cholesterol levels and hemopexin and hemoglobin, which were also negatively correlated with reticulocyte counts and LDH and bilirubin levels [29]. Together, these findings suggest that total cholesterol levels are related to pulmonary complications and hematological alterations in SCA.

We further observed that SCA individuals with increased HDL-C levels also had increased hemoglobin, hematocrit, total cholesterol, GGT, ferritin, and HbF levels, in addition to decreased RDW, VLDL-C, triglyceride, LDH, and NOm levels. Moreover, increased HDL-C levels were also associated with previous pain crises, and in the patients who reported painful episodes, HDL-C levels were also found to be correlated with hematological markers and HbF levels as well as total cholesterol levels. Additionally, total cholesterol and LDL-C levels were correlated with albumin levels. These results are consistent with a previous study that associated HDL-C levels with hemoglobin and hematocrit levels [30]. Likewise, in a study carried out by Zorca and colleagues that evaluated associations between lipid parameters and pulmonary hypertension, HDL-C levels were negatively correlated with LDH levels [20]. In the same study, triglyceride levels were correlated with markers of hemolysis, endothelial activation, and leukocyte counts [20]. These results reinforce the potential participation of HDL-C in modulating hemolysis and vascular dysfunction [31]. Albumin levels are thought to be increased in SCA individuals when compared to individuals with HbAS or HbAA genotype [32] and may be higher during hospitalization [33]. A previous study suggests that patients with chronic obstructive pulmonary disease present increased levels of ischemia-modified albumin as well as oxLDL, due to hypoxia, inflammation, and oxidative stress that these patients experience [34]. These mechanisms are also present in SCA pathogenesis; while the clinical presentation of SCA is highly variable, the most widely recognized clinical event is acute pain crisis driven by VO [35]. VO initiates a cascade of events, leading to tissue ischemia, which is responsible for the acute systemic vasoocclusive crises that frequently necessitate medical care for SCA patients [1]. Increased hematocrit levels are associated with blood rheology and red blood cell deformability, which can also contribute to VO and pain crises [1, 36]. Correspondingly, although the frequency of acute pain varies among SCA patients, it tends to be more frequent in patients presenting increased hematocrit and reduced HbF levels [37]. Moreover, besides the known anti-inflammatory and vasoprotective properties of HDL-C [14], recent data suggests the participation of HDL-C in the vascular environment with regard to hemolysis and anemia [31], which are important mechanisms underlying pain crises in SCA.

Decreased LDL-C levels were found in SCA individuals with a previous history of leg ulcers and increased in patients with a previous history of pneumonia, in addition to being associated with decreased MCV and MCH levels. The anemia presented by SCA patients is related to decreased red blood cell survival [38] and intravascular hemolysis [7], which creates a prooxidant and proinflammatory vascular milieu that contributes to endothelial dysfunction [39]. In this same vascular environment, LDL-C exerts a strong proinflammatory role [8], which could also contribute to SCA vasculopathy. This is further supported by evidence that LDL-C is also susceptible to oxidative modifications in SCA, based on the observation of increased binding of free heme to LDL-C fractions, which could favor the production of oxLDL [29]. Moreover, multiple vascular mechanisms have been attributed to the pathogenesis of leg ulcers in SCA, such as the physical obstruction caused by irreversibly sickled red blood cells, poor venous recirculation, bacterial infection, anemia, *in situ* thrombosis, and reduced NO bioavailability [40]. Patients with a previous history of leg ulcers exhibit elevated hemolytic laboratory parameters, increased uric acid, and decreased albumin levels [41]. Since the frequency of leg ulcers was associated with priapism and pulmonary hypertension, venous stasis could justify the causal relationship between pulmonary hypertension and leg ulcers, due to the overlapping of pathophysiological mechanisms [41]. Accordingly, the association between anemia and LDL-C levels suggests the vascular involvement of this molecule, which could also contribute to clinical manifestations.

Our findings stand in agreement with other studies that also reported decreased total cholesterol, HDL-C, and LDL-C levels among SCA individuals in comparison to HbAA individuals [19, 20, 25, 27]. It is thought that the hypocholesterolemia seen in SCA results from the augmented cholesterol utilization in erythropoiesis consequent to anemia and hemolysis. In addition, the occurrence of hypocholesterolemia in patients with nonhemolytic anemia suggests increased erythropoietic activity [17]. Moreover, it is also relevant to point that our investigation was carried out in a pediatric population of SCA patients, which could explain some discrepancies with data in the literatures, such as lack of association with different clinical manifestations; however, many of our results are in accordance with previous publications involving patients of a similar age, as well as adults [19, 20, 25]. The cross-sectional design of our study made it difficult to establish any causative roles for lipid parameters with regard to clinical manifestations in SCA, yet the relevant associations found herein will be useful in guiding further evaluations.

## 5. Conclusions

In summary, the present findings serve to affirm and extend the knowledge surrounding the abnormal lipid profile presented by SCA individuals in association with pain crises, leg ulcers, and pneumonia, in addition to upholding established correlations with laboratory markers of hemolysis and anemia.

## Figures and Tables

**Table 1:** Associations between lipid profile and laboratory parameters in SCA patients.

Laboratory parameters	Total cholesterol < 118 mg/dL (*N* = 64)	Total cholesterol ≥ 118 mg/dL (*N* = 62)	*p* value
Basophil (mL)	111.02 ± 107.40	76.69 ± 106.97	0.035
LDL-C (mg/dL)	47.98 ± 11.40	77.44 ± 18.63	0.000
VLDL-C (mg/dL)	19.41 ± 9.04	25.67 ± 12.42	0.000
Triglycerides (mg/dL)	94.17 ± 39.24	124.97 ± 55.95	0.000
Direct bilirubin (mg/dL)	0.38 ± 0.14	0.44 ± 0.16	0.021
Indirect bilirubin (mg/dL)	2.91 ± 1.74	2.32 ± 1.47	0.046
HbF (%)	10.37 ± 5.97	7.87 ± 5.14	0.015
	HDL‐C < 40 mg/dL (*N* = 89)	HDL‐C ≥ 40 mg/dL (*N* = 37)	
Hemoglobin (g/dL)	8.26 ± 0.99	8.99 ± 1.00	0.001
Hematocrit (%)	24.50 ± 3.25	26.85 ± 3.24	0.001∗
RDW (%)	23.14 ± 3.83	21.54 ± 3.52	0.027∗
Eosinophil (mL)	561 ± 548	326 ± 249	0.011
Total cholesterol (mg/dL)	118.27 ± 25.18	128.17 ± 22.96	0.025
VLDL-C (mg/dL)	24.32 ± 11.53	18.22 ± 9.65	0.001
Triglycerides (mg/dL)	119.43 ± 55.14	85.37 ± 25.01	0.001
GGT (U/L)	26.16 ± 23.31	30.7 ± 20.4	0.035
LDH (U/L)	1358.3 ± 1513.8	984.6 ± 355.8	0.006
Ferritin (*η*g/mL)	362.85 ± 506.69	482.64 ± 426.72	0.035
NOm (*μ*M)	26.38 ± 15.91	17.45 ± 5.52	0.001
HbF (%)	8.43 ± 5.60	10.73 ± 5.80	0.044
	LDL‐C < 58.8 mg/dL (*N* = 65)	LDL‐C ≥ 58.8 mg/dL (*N* = 61)	
MCV (fL)	94.63 ± 11.45	90.07 ± 11.44	0.028∗
MCH (*ρ*g/mL)	32.10 ± 3.91	30.52 ± 3.91	0.025∗

MCV: mean corpuscular volume; MCH: mean corpuscular hemoglobin; GGT: gamma-glutamyl transferase; RDW: red cell distribution width; LDH: lactate dehydrogenase; HbS: hemoglobin S; HbF: fetal hemoglobin; HDL-C: high-density lipoprotein cholesterol; LDL-C: low-density lipoprotein cholesterol; VLDL-C: very low-density lipoprotein cholesterol; NOm: nitric oxide metabolites. *p* value obtained using Mann-Whitney *U* test. ^∗^*p* value obtained using independent *t*-test.

**Table 2:** Frequency of clinical manifestations associated with lipid parameters in SCA patients.

Clinical data	Lipid parameters		*p* value
Pneumonia + (*N* = 69)	Total cholesterol < 118 mg/dL	Total cholesterol ≥ 118 mg/dL	0.007
	27 (39%)	42 (61%)	
Pneumonia + (*N* = 69)	LDL‐C < 58.8 mg/dL	LDL‐C ≥ 58.8 mg/dL	0.048
	29 (42%)	40 (58%)	

Data comparisons performed using Fisher's exact test.

## Data Availability

All relevant data are within the paper.

## Abbreviations

GGT: Gamma-glutamyl transferase
HbF: Fetal hemoglobin
HbS: Hemoglobin S
HDL-C: High-density lipoprotein cholesterol
LDH: Lactate dehydrogenase
LDL-C: Low-density lipoprotein cholesterol
MCH: Mean corpuscular hemoglobin
MCHC: Mean corpuscular hemoglobin concentration
MCV: Mean corpuscular volume
NO: Nitric oxide
RDW: Red cell distribution width
SCA: Sickle cell anemia
VLDL-C: Very low-density lipoprotein cholesterol
VO: Vasoocclusion.
